# (2-Chloro­pyrimidin-4-yl)ferrocene

**DOI:** 10.1107/S1600536814004917

**Published:** 2014-03-12

**Authors:** Guo-Qing Shi, Yang Li, Xiang Gao, Long-Fei Hao, Wen-en Zhao

**Affiliations:** a School of Chemical Engineering and Energy, Zhengzhou University, Henan, Zhengzhou 450001, People’s Republic of China; b School of Food and Bioengineering, Zhengzhou University of Light Industry, Henan, Zhengzhou 450001, People’s Republic of China; c College of Chemistry and Molecular Engineering, Zhengzhou University, Henan, Zhengzhou 450001, People’s Republic of China

## Abstract

In the title compound, [Fe(C_5_H_5_)(C_9_H_6_ClN_2_)], the two cyclo­penta­dienyl rings are almost parallel, subtending a dihedral angle of 3.01 (5)°. The dihedral angle between the substituted cyclo­penta­dienyl ring and the pyrimidinyl ring is 12.02 (1)°. The conformation of the two cyclopentadienyl rings in the ferrocenyl moiety is eclipsed.

## Related literature   

For pyrimidinyl derivatives, see: Chinchilla *et al.* (2004 ▶); Walker *et al.* (2009 ▶). For ferrocenyl pyrimidines, see: Xu *et al.* (2009 ▶, 2010 ▶). For the synthesis of the title compound, see: Xu *et al.* (2014 ▶). 
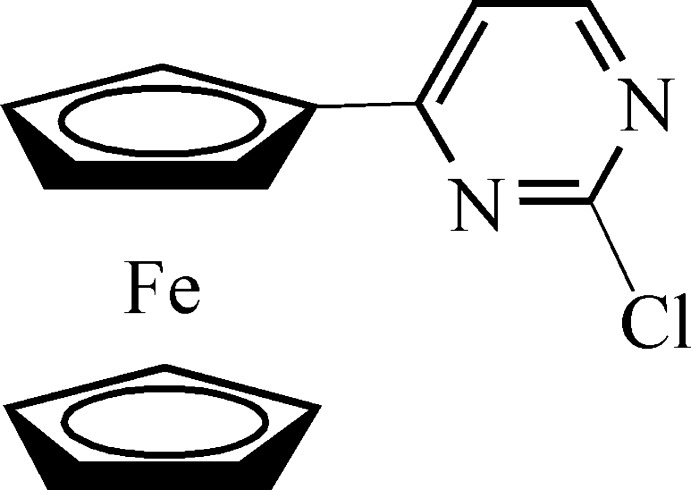


## Experimental   

### 

#### Crystal data  

[Fe(C_5_H_5_)(C_9_H_6_ClN_2_)]
*M* *_r_* = 298.55
Monoclinic, 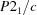

*a* = 10.5064 (18) Å
*b* = 10.2684 (17) Å
*c* = 11.843 (2) Å
β = 108.580 (2)°
*V* = 1211.1 (4) Å^3^
*Z* = 4
Mo *K*α radiation
μ = 1.45 mm^−1^
*T* = 296 K
0.43 × 0.15 × 0.12 mm

#### Data collection  

Bruker SMART APEX CCD area-detector diffractometer
Absorption correction: multi-scan (*SADABS*; Bruker, 2004 ▶) *T*_min_ = 0.575, *T*_max_ = 0.846
8932 measured reflections
2251 independent reflections
1777 reflections with *I* > 2σ(*I*)
*R*_int_ = 0.034

#### Refinement  

*R*[*F* ^2^ > 2σ(*F* ^2^)] = 0.032
*wR*(*F* ^2^) = 0.111
*S* = 0.73
2251 reflections
163 parameters
H-atom parameters constrained
Δρ_max_ = 0.28 e Å^−3^
Δρ_min_ = −0.23 e Å^−3^

### 

Data collection: *APEX2* (Bruker, 2004 ▶); cell refinement: *SAINT* (Bruker, 2004 ▶); data reduction: *SAINT*; program(s) used to solve structure: *SHELXS97* (Sheldrick, 2008 ▶); program(s) used to refine structure: *SHELXL97* (Sheldrick, 2008 ▶); molecular graphics: *SHELXTL* (Sheldrick, 2008 ▶); software used to prepare material for publication: *SHELXL97*.

## Supplementary Material

Crystal structure: contains datablock(s) global, I. DOI: 10.1107/S1600536814004917/fj2665sup1.cif

Structure factors: contains datablock(s) I. DOI: 10.1107/S1600536814004917/fj2665Isup2.hkl

CCDC reference: 989734

Additional supporting information:  crystallographic information; 3D view; checkCIF report

## Figures and Tables

**Fig. 1.**
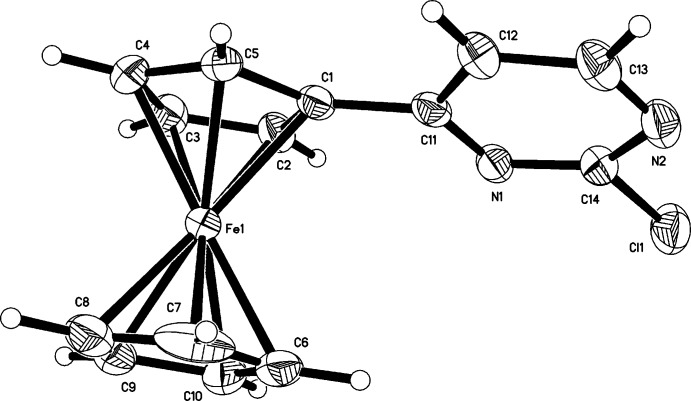
The molecular structure of the title compound with displacement ellipsoids at the 30% probability level.

## supplementary crystallographic information

### 1. Comment

Pyrimidines are widespread heterocyclic motifs found in many natural products and pharmaceuticals (Chinchilla *et al.*, 2004; Walker *et al.*, 2009). In addition, ferrocenyl pyrimidines as ligands are used in organometallic catalysis (Xu *et al.*, 2009,2010). Here we report the crystal structure of the title compound, obtained from the *via* the coupling reaction of chloromercuriferrocene and 4,6-dichloropyrimidine.

A view on the molecular structure of the title compound is given in the figure 1. The two cyclopentadienyl rings are almost parallel with dihedral angles of 3.01 (5)°. The dihedral angle between the substituted cyclopentadienyl and pyrimidinyl ring is 12.02 (1)°. The nitrogen and chlorine atoms of pyrimidinyl ring do not participate in hydrogen bond.

### 2. Experimental

The title compound was prepared as described in literature (Xu *et al.* 2014) and recrystallized from dichloromethane/petroleum ether solution at room temperature to give the desired crystals suitable for single-crystal X-ray diffraction.

### 3. Refinement

H atoms attached to C atoms of the title compound were placed in geometrically idealized positions and treated as riding with C—H distances constrained to 0.93–0.96 Å, and with *U*ĩso~(H)=1.2Ueq(C).

### Crystal data

**Table:**

[Fe(C_5_H_5_)(C_9_H_6_ClN_2_)]	*F*(000) = 608
*M**_r_* = 298.55	*D*_x_ = 1.637 Mg m^−^^3^
Monoclinic, *P*2_1_/*c*	Mo *K*α radiation, λ = 0.71073 Å
Hall symbol: -P 2ybc	Cell parameters from 2413 reflections
*a* = 10.5064 (18) Å	θ = 2.7–24.7°
*b* = 10.2684 (17) Å	µ = 1.45 mm^−^^1^
*c* = 11.843 (2) Å	*T* = 296 K
β = 108.580 (2)°	Block, red
*V* = 1211.1 (4) Å^3^	0.43 × 0.15 × 0.12 mm
*Z* = 4	

### Data collection

**Table:**

Bruker SMART APEX CCD area-detector diffractometer	2251 independent reflections
Radiation source: fine-focus sealed tube	1777 reflections with *I* > 2σ(*I*)
Graphite monochromator	*R*_int_ = 0.034
phi and ω scans	θ_max_ = 25.5°, θ_min_ = 2.7°
Absorption correction: multi-scan (*SADABS*; Bruker, 2004)	*h* = −12→12
*T*_min_ = 0.575, *T*_max_ = 0.846	*k* = −12→12
8932 measured reflections	*l* = −14→14

### Refinement

**Table:**

Refinement on *F*^2^	Primary atom site location: structure-invariant direct methods
Least-squares matrix: full	Secondary atom site location: difference Fourier map
*R*[*F*^2^ > 2σ(*F*^2^)] = 0.032	Hydrogen site location: inferred from neighbouring sites
*wR*(*F*^2^) = 0.111	H-atom parameters constrained
*S* = 0.73	*w* = 1/[σ^2^(*F*_o_^2^) + (0.1*P*)^2^ + 1.*P*] where *P* = (*F*_o_^2^ + 2*F*_c_^2^)/3
2251 reflections	(Δ/σ)_max_ = 0.001
163 parameters	Δρ_max_ = 0.28 e Å^−^^3^
0 restraints	Δρ_min_ = −0.23 e Å^−^^3^

### Special details

**Table:**

Geometry. All e.s.d.'s (except the e.s.d. in the dihedral angle between two l.s. planes)are estimated using the full covariance matrix. The cell e.s.d.'s are takeninto account individually in the estimation of e.s.d.'s in distances, anglesand torsion angles; correlations between e.s.d.'s in cell parameters are onlyused when they are defined by crystal symmetry. An approximate (isotropic)treatment of cell e.s.d.'s is used for estimating e.s.d.'s involving l.s. planes.
Refinement. Refinement of *F*^2^ against ALL reflections. The weighted *R*-factor *wR* and goodness of fit *S* are based on *F*^2^, conventional *R*-factors *R* are based on *F*, with *F* set to zero for negative *F*^2^. The threshold expression of *F*^2^ > σ(*F*^2^) is used only for calculating *R*-factors(gt) *etc*. and is not relevant to the choice of reflections for refinement. *R*-factors based on *F*^2^ are statistically about twice as large as those based on *F*, and *R*- factors based on ALL data will be even larger.

### Fractional atomic coordinates and isotropic or equivalent isotropic displacement parameters (Å^2^)

**Table:**

	*x*	*y*	*z*	*U*_iso_*/*U*_eq_	
Fe1	0.83734 (4)	0.37324 (4)	0.13102 (3)	0.03675 (17)	
Cl1	0.30992 (10)	0.08466 (10)	0.06208 (8)	0.0682 (3)	
N1	0.4864 (2)	0.2434 (2)	0.0285 (2)	0.0417 (6)	
N2	0.3649 (3)	0.1085 (3)	−0.1350 (2)	0.0508 (7)	
C1	0.6492 (3)	0.4015 (3)	0.0148 (3)	0.0385 (6)	
C2	0.6641 (3)	0.4632 (3)	0.1266 (3)	0.0437 (7)	
H2	0.6107	0.4447	0.1791	0.052*	
C3	0.7683 (3)	0.5561 (3)	0.1477 (3)	0.0493 (8)	
H3	0.8004	0.6120	0.2182	0.059*	
C4	0.8204 (3)	0.5527 (3)	0.0520 (3)	0.0489 (8)	
H4	0.8948	0.6057	0.0444	0.059*	
C5	0.7475 (3)	0.4585 (3)	−0.0309 (3)	0.0419 (7)	
H5	0.7626	0.4353	−0.1059	0.050*	
C6	0.8598 (4)	0.1813 (3)	0.1773 (4)	0.0639 (10)	
H6	0.7911	0.1132	0.1529	0.077*	
C7	0.9520 (5)	0.2188 (5)	0.1171 (4)	0.0812 (14)	
H7	0.9582	0.1812	0.0430	0.097*	
C8	1.0339 (4)	0.3189 (5)	0.1849 (4)	0.0733 (12)	
H8	1.1066	0.3637	0.1654	0.088*	
C9	0.9945 (4)	0.3419 (4)	0.2828 (3)	0.0655 (11)	
H9	1.0339	0.4064	0.3451	0.079*	
C10	0.8885 (4)	0.2581 (4)	0.2782 (3)	0.0583 (9)	
H10	0.8409	0.2548	0.3372	0.070*	
C11	0.5533 (3)	0.2986 (3)	−0.0397 (2)	0.0389 (6)	
C12	0.5267 (3)	0.2604 (3)	−0.1578 (3)	0.0457 (7)	
H12	0.5714	0.2980	−0.2059	0.055*	
C13	0.4320 (3)	0.1651 (3)	−0.2007 (3)	0.0511 (8)	
H13	0.4136	0.1385	−0.2793	0.061*	
C14	0.3983 (3)	0.1538 (3)	−0.0259 (3)	0.0464 (7)	

### Atomic displacement parameters (Å^2^)

**Table:**

	*U*^11^	*U*^22^	*U*^33^	*U*^12^	*U*^13^	*U*^23^
Fe1	0.0331 (3)	0.0351 (3)	0.0377 (3)	−0.00076 (15)	0.00512 (18)	0.00464 (15)
Cl1	0.0639 (6)	0.0770 (6)	0.0607 (6)	−0.0252 (5)	0.0156 (4)	0.0025 (5)
N1	0.0371 (12)	0.0417 (13)	0.0414 (13)	0.0004 (10)	0.0053 (10)	−0.0030 (10)
N2	0.0431 (15)	0.0503 (15)	0.0523 (16)	−0.0046 (11)	0.0059 (13)	−0.0119 (12)
C1	0.0355 (15)	0.0343 (13)	0.0390 (15)	0.0042 (11)	0.0024 (12)	0.0005 (11)
C2	0.0378 (15)	0.0423 (16)	0.0481 (16)	0.0026 (13)	0.0095 (13)	−0.0074 (13)
C3	0.0484 (18)	0.0362 (15)	0.0559 (18)	−0.0029 (13)	0.0063 (15)	−0.0104 (13)
C4	0.0500 (18)	0.0353 (15)	0.0556 (19)	−0.0059 (13)	0.0088 (15)	0.0069 (13)
C5	0.0450 (16)	0.0376 (15)	0.0387 (14)	0.0016 (13)	0.0074 (12)	0.0071 (12)
C6	0.062 (2)	0.0346 (17)	0.075 (2)	0.0047 (15)	−0.0064 (19)	0.0087 (16)
C7	0.099 (3)	0.087 (3)	0.055 (2)	0.062 (3)	0.021 (2)	0.015 (2)
C8	0.0395 (19)	0.086 (3)	0.090 (3)	0.0114 (19)	0.0146 (19)	0.040 (3)
C9	0.057 (2)	0.057 (2)	0.060 (2)	−0.0097 (17)	−0.0145 (18)	0.0175 (17)
C10	0.062 (2)	0.059 (2)	0.0475 (18)	−0.0065 (16)	0.0076 (16)	0.0178 (16)
C11	0.0343 (14)	0.0360 (14)	0.0399 (15)	0.0084 (11)	0.0027 (12)	0.0004 (11)
C12	0.0400 (16)	0.0533 (18)	0.0403 (16)	−0.0010 (13)	0.0081 (13)	−0.0074 (13)
C13	0.0447 (18)	0.0585 (19)	0.0440 (17)	0.0015 (15)	0.0056 (14)	−0.0140 (15)
C14	0.0396 (16)	0.0430 (16)	0.0508 (18)	−0.0013 (13)	0.0064 (14)	0.0020 (13)

### Geometric parameters (Å, º)

**Table:**

Fe1—C2	2.027 (3)	C3—C4	1.408 (5)
Fe1—C7	2.029 (4)	C3—H3	0.9800
Fe1—C10	2.032 (3)	C4—C5	1.416 (4)
Fe1—C8	2.036 (4)	C4—H4	0.9800
Fe1—C1	2.038 (3)	C5—H5	0.9800
Fe1—C6	2.040 (3)	C6—C10	1.383 (5)
Fe1—C9	2.042 (3)	C6—C7	1.426 (6)
Fe1—C3	2.045 (3)	C6—H6	0.9800
Fe1—C5	2.045 (3)	C7—C8	1.415 (6)
Fe1—C4	2.049 (3)	C7—H7	0.9800
Cl1—C14	1.751 (3)	C8—C9	1.370 (6)
N1—C14	1.319 (4)	C8—H8	0.9800
N1—C11	1.352 (4)	C9—C10	1.395 (5)
N2—C14	1.313 (4)	C9—H9	0.9800
N2—C13	1.337 (4)	C10—H10	0.9800
C1—C2	1.430 (4)	C11—C12	1.394 (4)
C1—C5	1.436 (4)	C12—C13	1.372 (4)
C1—C11	1.461 (4)	C12—H12	0.9300
C2—C3	1.413 (4)	C13—H13	0.9300
C2—H2	0.9800		
			
C2—Fe1—C7	154.86 (19)	C4—C3—Fe1	70.02 (17)
C2—Fe1—C10	106.10 (15)	C2—C3—Fe1	69.01 (16)
C7—Fe1—C10	67.27 (16)	C4—C3—H3	125.7
C2—Fe1—C8	161.11 (18)	C2—C3—H3	125.7
C7—Fe1—C8	40.73 (19)	Fe1—C3—H3	125.7
C10—Fe1—C8	66.88 (16)	C3—C4—C5	108.2 (3)
C2—Fe1—C1	41.18 (12)	C3—C4—Fe1	69.75 (17)
C7—Fe1—C1	121.99 (16)	C5—C4—Fe1	69.62 (16)
C10—Fe1—C1	126.63 (14)	C3—C4—H4	125.9
C8—Fe1—C1	156.89 (17)	C5—C4—H4	125.9
C2—Fe1—C6	118.27 (15)	Fe1—C4—H4	125.9
C7—Fe1—C6	41.04 (17)	C4—C5—C1	108.2 (3)
C10—Fe1—C6	39.72 (15)	C4—C5—Fe1	69.89 (17)
C8—Fe1—C6	68.40 (16)	C1—C5—Fe1	69.14 (15)
C1—Fe1—C6	108.89 (13)	C4—C5—H5	125.9
C2—Fe1—C9	124.24 (16)	C1—C5—H5	125.9
C7—Fe1—C9	67.28 (18)	Fe1—C5—H5	125.9
C10—Fe1—C9	40.06 (15)	C10—C6—C7	106.4 (3)
C8—Fe1—C9	39.26 (17)	C10—C6—Fe1	69.84 (19)
C1—Fe1—C9	162.82 (16)	C7—C6—Fe1	69.1 (2)
C6—Fe1—C9	67.62 (15)	C10—C6—H6	126.8
C2—Fe1—C3	40.60 (12)	C7—C6—H6	126.8
C7—Fe1—C3	164.41 (19)	Fe1—C6—H6	126.8
C10—Fe1—C3	117.13 (15)	C8—C7—C6	107.5 (4)
C8—Fe1—C3	125.25 (17)	C8—C7—Fe1	69.9 (2)
C1—Fe1—C3	68.70 (12)	C6—C7—Fe1	69.9 (2)
C6—Fe1—C3	151.12 (16)	C8—C7—H7	126.3
C9—Fe1—C3	105.78 (15)	C6—C7—H7	126.3
C2—Fe1—C5	68.79 (13)	Fe1—C7—H7	126.3
C7—Fe1—C5	111.52 (14)	C9—C8—C7	108.2 (4)
C10—Fe1—C5	166.08 (13)	C9—C8—Fe1	70.6 (2)
C8—Fe1—C5	122.01 (15)	C7—C8—Fe1	69.4 (2)
C1—Fe1—C5	41.17 (12)	C9—C8—H8	125.9
C6—Fe1—C5	130.18 (14)	C7—C8—H8	125.9
C9—Fe1—C5	153.51 (14)	Fe1—C8—H8	125.9
C3—Fe1—C5	68.03 (12)	C8—C9—C10	108.3 (4)
C2—Fe1—C4	68.38 (13)	C8—C9—Fe1	70.1 (2)
C7—Fe1—C4	129.28 (17)	C10—C9—Fe1	69.58 (19)
C10—Fe1—C4	151.17 (14)	C8—C9—H9	125.9
C8—Fe1—C4	108.75 (15)	C10—C9—H9	125.9
C1—Fe1—C4	68.83 (12)	Fe1—C9—H9	125.9
C6—Fe1—C4	167.93 (16)	C6—C10—C9	109.7 (3)
C9—Fe1—C4	118.17 (14)	C6—C10—Fe1	70.44 (19)
C3—Fe1—C4	40.24 (13)	C9—C10—Fe1	70.36 (19)
C5—Fe1—C4	40.49 (12)	C6—C10—H10	125.2
C14—N1—C11	114.7 (3)	C9—C10—H10	125.2
C14—N2—C13	113.2 (3)	Fe1—C10—H10	125.2
C2—C1—C5	106.8 (3)	N1—C11—C12	120.4 (3)
C2—C1—C11	125.9 (3)	N1—C11—C1	117.1 (2)
C5—C1—C11	127.3 (3)	C12—C11—C1	122.5 (3)
C2—C1—Fe1	69.01 (16)	C13—C12—C11	117.5 (3)
C5—C1—Fe1	69.69 (16)	C13—C12—H12	121.2
C11—C1—Fe1	125.37 (19)	C11—C12—H12	121.2
C3—C2—C1	108.3 (3)	N2—C13—C12	123.3 (3)
C3—C2—Fe1	70.38 (17)	N2—C13—H13	118.3
C1—C2—Fe1	69.81 (16)	C12—C13—H13	118.3
C3—C2—H2	125.9	N2—C14—N1	130.9 (3)
C1—C2—H2	125.9	N2—C14—Cl1	114.6 (2)
Fe1—C2—H2	125.9	N1—C14—Cl1	114.5 (2)
C4—C3—C2	108.6 (3)		
			
C7—Fe1—C1—C2	155.1 (2)	C8—Fe1—C6—C10	79.4 (3)
C10—Fe1—C1—C2	71.0 (2)	C1—Fe1—C6—C10	−125.1 (2)
C8—Fe1—C1—C2	−169.3 (4)	C9—Fe1—C6—C10	36.9 (2)
C6—Fe1—C1—C2	111.7 (2)	C3—Fe1—C6—C10	−45.2 (4)
C9—Fe1—C1—C2	36.5 (5)	C5—Fe1—C6—C10	−166.3 (2)
C3—Fe1—C1—C2	−37.63 (18)	C4—Fe1—C6—C10	157.9 (6)
C5—Fe1—C1—C2	−118.2 (2)	C2—Fe1—C6—C7	161.4 (2)
C4—Fe1—C1—C2	−80.94 (19)	C10—Fe1—C6—C7	−117.5 (3)
C2—Fe1—C1—C5	118.2 (2)	C8—Fe1—C6—C7	−38.1 (3)
C7—Fe1—C1—C5	−86.7 (2)	C1—Fe1—C6—C7	117.4 (2)
C10—Fe1—C1—C5	−170.79 (19)	C9—Fe1—C6—C7	−80.6 (3)
C8—Fe1—C1—C5	−51.1 (4)	C3—Fe1—C6—C7	−162.7 (3)
C6—Fe1—C1—C5	−130.1 (2)	C5—Fe1—C6—C7	76.2 (3)
C9—Fe1—C1—C5	154.7 (4)	C4—Fe1—C6—C7	40.4 (7)
C3—Fe1—C1—C5	80.55 (18)	C10—C6—C7—C8	−0.2 (4)
C4—Fe1—C1—C5	37.24 (17)	Fe1—C6—C7—C8	60.0 (2)
C2—Fe1—C1—C11	−119.8 (3)	C10—C6—C7—Fe1	−60.2 (2)
C7—Fe1—C1—C11	35.2 (3)	C2—Fe1—C7—C8	−159.8 (3)
C10—Fe1—C1—C11	−48.8 (3)	C10—Fe1—C7—C8	−80.4 (3)
C8—Fe1—C1—C11	70.9 (5)	C1—Fe1—C7—C8	159.5 (2)
C6—Fe1—C1—C11	−8.2 (3)	C6—Fe1—C7—C8	−118.4 (3)
C9—Fe1—C1—C11	−83.3 (5)	C9—Fe1—C7—C8	−36.9 (2)
C3—Fe1—C1—C11	−157.5 (3)	C3—Fe1—C7—C8	29.3 (7)
C5—Fe1—C1—C11	122.0 (3)	C5—Fe1—C7—C8	114.5 (2)
C4—Fe1—C1—C11	159.2 (3)	C4—Fe1—C7—C8	71.7 (3)
C5—C1—C2—C3	0.4 (3)	C2—Fe1—C7—C6	−41.4 (4)
C11—C1—C2—C3	179.3 (3)	C10—Fe1—C7—C6	37.9 (2)
Fe1—C1—C2—C3	60.1 (2)	C8—Fe1—C7—C6	118.4 (3)
C5—C1—C2—Fe1	−59.72 (19)	C1—Fe1—C7—C6	−82.2 (2)
C11—C1—C2—Fe1	119.2 (3)	C9—Fe1—C7—C6	81.5 (2)
C7—Fe1—C2—C3	−176.3 (3)	C3—Fe1—C7—C6	147.7 (5)
C10—Fe1—C2—C3	113.1 (2)	C5—Fe1—C7—C6	−127.1 (2)
C8—Fe1—C2—C3	47.9 (5)	C4—Fe1—C7—C6	−169.9 (2)
C1—Fe1—C2—C3	−119.1 (3)	C6—C7—C8—C9	0.2 (4)
C6—Fe1—C2—C3	154.2 (2)	Fe1—C7—C8—C9	60.2 (3)
C9—Fe1—C2—C3	73.2 (2)	C6—C7—C8—Fe1	−60.0 (2)
C5—Fe1—C2—C3	−80.56 (19)	C2—Fe1—C8—C9	34.0 (6)
C4—Fe1—C2—C3	−36.93 (18)	C7—Fe1—C8—C9	−119.1 (3)
C7—Fe1—C2—C1	−57.2 (4)	C10—Fe1—C8—C9	−37.6 (2)
C10—Fe1—C2—C1	−127.83 (19)	C1—Fe1—C8—C9	−168.3 (3)
C8—Fe1—C2—C1	167.0 (4)	C6—Fe1—C8—C9	−80.6 (3)
C6—Fe1—C2—C1	−86.8 (2)	C3—Fe1—C8—C9	70.2 (3)
C9—Fe1—C2—C1	−167.72 (18)	C5—Fe1—C8—C9	154.6 (2)
C3—Fe1—C2—C1	119.1 (3)	C4—Fe1—C8—C9	111.9 (2)
C5—Fe1—C2—C1	38.49 (17)	C2—Fe1—C8—C7	153.0 (4)
C4—Fe1—C2—C1	82.13 (18)	C10—Fe1—C8—C7	81.5 (3)
C1—C2—C3—C4	−0.8 (3)	C1—Fe1—C8—C7	−49.2 (5)
Fe1—C2—C3—C4	59.0 (2)	C6—Fe1—C8—C7	38.4 (2)
C1—C2—C3—Fe1	−59.8 (2)	C9—Fe1—C8—C7	119.1 (4)
C2—Fe1—C3—C4	−120.2 (3)	C3—Fe1—C8—C7	−170.7 (2)
C7—Fe1—C3—C4	53.9 (6)	C5—Fe1—C8—C7	−86.4 (3)
C10—Fe1—C3—C4	156.68 (19)	C4—Fe1—C8—C7	−129.1 (2)
C8—Fe1—C3—C4	76.9 (2)	C7—C8—C9—C10	−0.1 (4)
C1—Fe1—C3—C4	−81.99 (19)	Fe1—C8—C9—C10	59.3 (3)
C6—Fe1—C3—C4	−172.7 (3)	C7—C8—C9—Fe1	−59.5 (2)
C9—Fe1—C3—C4	115.2 (2)	C2—Fe1—C9—C8	−167.4 (2)
C5—Fe1—C3—C4	−37.55 (17)	C7—Fe1—C9—C8	38.2 (3)
C7—Fe1—C3—C2	174.1 (5)	C10—Fe1—C9—C8	119.4 (4)
C10—Fe1—C3—C2	−83.2 (2)	C1—Fe1—C9—C8	164.3 (4)
C8—Fe1—C3—C2	−162.9 (2)	C6—Fe1—C9—C8	82.8 (3)
C1—Fe1—C3—C2	38.16 (18)	C3—Fe1—C9—C8	−127.0 (3)
C6—Fe1—C3—C2	−52.6 (3)	C5—Fe1—C9—C8	−54.8 (4)
C9—Fe1—C3—C2	−124.7 (2)	C4—Fe1—C9—C8	−85.5 (3)
C5—Fe1—C3—C2	82.60 (19)	C2—Fe1—C9—C10	73.2 (3)
C4—Fe1—C3—C2	120.2 (3)	C7—Fe1—C9—C10	−81.2 (3)
C2—C3—C4—C5	0.8 (3)	C8—Fe1—C9—C10	−119.4 (4)
Fe1—C3—C4—C5	59.2 (2)	C1—Fe1—C9—C10	44.9 (6)
C2—C3—C4—Fe1	−58.4 (2)	C6—Fe1—C9—C10	−36.6 (2)
C2—Fe1—C4—C3	37.25 (17)	C3—Fe1—C9—C10	113.6 (2)
C7—Fe1—C4—C3	−163.7 (2)	C5—Fe1—C9—C10	−174.2 (3)
C10—Fe1—C4—C3	−46.9 (4)	C4—Fe1—C9—C10	155.1 (2)
C8—Fe1—C4—C3	−122.8 (2)	C7—C6—C10—C9	0.1 (4)
C1—Fe1—C4—C3	81.64 (19)	Fe1—C6—C10—C9	−59.6 (3)
C6—Fe1—C4—C3	162.9 (6)	C7—C6—C10—Fe1	59.7 (2)
C9—Fe1—C4—C3	−81.1 (2)	C8—C9—C10—C6	0.0 (4)
C5—Fe1—C4—C3	119.5 (3)	Fe1—C9—C10—C6	59.7 (2)
C2—Fe1—C4—C5	−82.23 (19)	C8—C9—C10—Fe1	−59.6 (3)
C7—Fe1—C4—C5	76.8 (3)	C2—Fe1—C10—C6	115.1 (2)
C10—Fe1—C4—C5	−166.4 (3)	C7—Fe1—C10—C6	−39.1 (3)
C8—Fe1—C4—C5	117.7 (2)	C8—Fe1—C10—C6	−83.5 (3)
C1—Fe1—C4—C5	−37.85 (18)	C1—Fe1—C10—C6	74.7 (3)
C6—Fe1—C4—C5	43.5 (7)	C9—Fe1—C10—C6	−120.4 (3)
C9—Fe1—C4—C5	159.5 (2)	C3—Fe1—C10—C6	157.4 (2)
C3—Fe1—C4—C5	−119.5 (3)	C5—Fe1—C10—C6	48.7 (7)
C3—C4—C5—C1	−0.6 (3)	C4—Fe1—C10—C6	−170.6 (3)
Fe1—C4—C5—C1	58.73 (19)	C2—Fe1—C10—C9	−124.5 (3)
C3—C4—C5—Fe1	−59.3 (2)	C7—Fe1—C10—C9	81.2 (3)
C2—C1—C5—C4	0.1 (3)	C8—Fe1—C10—C9	36.8 (3)
C11—C1—C5—C4	−178.8 (3)	C1—Fe1—C10—C9	−164.9 (2)
Fe1—C1—C5—C4	−59.2 (2)	C6—Fe1—C10—C9	120.4 (3)
C2—C1—C5—Fe1	59.28 (19)	C3—Fe1—C10—C9	−82.3 (3)
C11—C1—C5—Fe1	−119.6 (3)	C5—Fe1—C10—C9	169.1 (5)
C2—Fe1—C5—C4	81.1 (2)	C4—Fe1—C10—C9	−50.2 (4)
C7—Fe1—C5—C4	−125.9 (2)	C14—N1—C11—C12	0.3 (4)
C10—Fe1—C5—C4	151.9 (6)	C14—N1—C11—C1	178.1 (3)
C8—Fe1—C5—C4	−81.5 (3)	C2—C1—C11—N1	−10.2 (4)
C1—Fe1—C5—C4	119.6 (3)	C5—C1—C11—N1	168.4 (3)
C6—Fe1—C5—C4	−169.2 (2)	Fe1—C1—C11—N1	78.1 (3)
C9—Fe1—C5—C4	−43.9 (4)	C2—C1—C11—C12	167.5 (3)
C3—Fe1—C5—C4	37.33 (18)	C5—C1—C11—C12	−13.8 (4)
C2—Fe1—C5—C1	−38.51 (17)	Fe1—C1—C11—C12	−104.2 (3)
C7—Fe1—C5—C1	114.5 (2)	N1—C11—C12—C13	−0.6 (4)
C10—Fe1—C5—C1	32.2 (6)	C1—C11—C12—C13	−178.2 (3)
C8—Fe1—C5—C1	158.9 (2)	C14—N2—C13—C12	0.4 (5)
C6—Fe1—C5—C1	71.2 (2)	C11—C12—C13—N2	0.2 (5)
C9—Fe1—C5—C1	−163.6 (3)	C13—N2—C14—N1	−0.8 (5)
C3—Fe1—C5—C1	−82.32 (18)	C13—N2—C14—Cl1	178.7 (2)
C4—Fe1—C5—C1	−119.6 (3)	C11—N1—C14—N2	0.4 (5)
C2—Fe1—C6—C10	−81.1 (2)	C11—N1—C14—Cl1	−179.0 (2)
C7—Fe1—C6—C10	117.5 (3)		
